# Recurrent Desmoid Tumor of the Buttock in a Preadolescent Child

**Published:** 2012-03-01

**Authors:** Yogesh Kumar Sarin

**Affiliations:** Department of Pediatric Surgery, Maulana Azad Medical College, New Delhi-110002

**Dear Sir**

This is in reference to our article published before [1]. On 1 year follow up, the patient had local recurrence measuring 8 cm X 5 cm infero-laterally to the previous scar. MRI of the local region showed a large well defined heterogeneously enhancing soft tissue mass in subcutaneous layers involving ilio-tabial tract with areas of deep multiple recurrences (Fig. 1). There was no evidence of metastasis. Local excision was done. Histopathology was identical as before- the desmoid tumor. The surgical margins were free; the mass was surrounded with fibrocollagenous tissue. Keeping in view the unresectable deep recurrences, he was administered radiotherapy locally. MRI done after 6 weeks of radiotherapy did not show any residual disease. The child is under close follow up.

**Figure:**
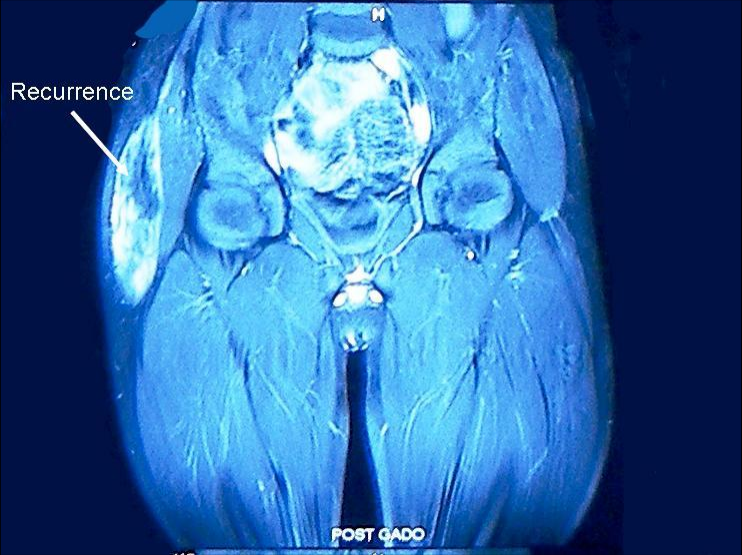
Figure 1: MRI showing large subcutaneous local recurrence and multiple small deep recurrences.

Even after multiple recurrences, successful salvage is achievable, particularly when high-dose focal radiotherapy is incorporated [2]. It would also be imperative to mention role of intra-operative electron radiotherapy (IOERT), followed by moderate doses of external beam radiotherapy (EBRT) after organ-sparing surgery, in patients with primary or recurrent aggressive fibromatosis. Introduction of IOERT into a multimodal treatment approach in patients with aggressive fibromatosis is feasible with low toxicity and is known to yield good local control rates even in patients with microscopical or gross residual disease [3]. Such facility is however unavailable in our setup. In our case, the hip joint was not involved. A case of aggressive pediatric hip fibromatosis with severe joint destruction has been reported recently [4], and we would like to prepare ourselves for such an eventuality. 

## Footnotes

**Source of Support:** Nil

**Conflict of Interest:** None declared
